# Regional differences in clonal Japanese knotweed revealed by chemometrics-linked attenuated total reflection Fourier-transform infrared spectroscopy

**DOI:** 10.1186/s12870-021-03293-y

**Published:** 2021-11-09

**Authors:** Claire A. Holden, Camilo L. M. Morais, Jane E. Taylor, Francis L. Martin, Paul Beckett, Martin McAinsh

**Affiliations:** 1 Lancaster Environment Centre, Lancaster University, Lancaster, UK; 2 School of Pharmacy and Biomedical Sciences, University of Central Lancashire, Preston, UK; 3 Biocel Ltd, Hull, HU10 7TS UK; 4 Phlorum Ltd, Brighton, UK

**Keywords:** Ecosystem, Epigenomics, FTIR spectroscopy, Invasive species, Japanese knotweed, Physiological adaptation, Plants, Principal component analysis, Spectrum analysis, Support vector machine

## Abstract

**Background:**

Japanese knotweed (*R. japonica var japonica)* is one of the world’s 100 worst invasive species, causing crop losses, damage to infrastructure, and erosion of ecosystem services. In the UK, this species is an all-female clone, which spreads by vegetative reproduction. Despite this genetic continuity, Japanese knotweed can colonise a wide variety of environmental habitats. However, little is known about the phenotypic plasticity responsible for the ability of Japanese knotweed to invade and thrive in such diverse habitats. We have used attenuated total reflection Fourier-transform infrared (ATR-FTIR) spectroscopy, in which the spectral fingerprint generated allows subtle differences in composition to be clearly visualized, to examine regional differences in clonal Japanese knotweed.

**Results:**

We have shown distinct differences in the spectral fingerprint region (1800–900 cm^− 1^) of Japanese knotweed from three different regions in the UK that were sufficient to successfully identify plants from different geographical regions with high accuracy using support vector machine (SVM) chemometrics.

**Conclusions:**

These differences were not correlated with environmental variations between regions, raising the possibility that epigenetic modifications may contribute to the phenotypic plasticity responsible for the ability of *R. japonica* to invade and thrive in such diverse habitats.

**Supplementary Information:**

The online version contains supplementary material available at 10.1186/s12870-021-03293-y.

## Background

Invasive Alien Species (IAS) constitute a major threat to global biodiversity [1]. Japanese knotweed (*Reynoutria japonica var. japonica*) [2, 3] is particularly invasive throughout North America, Europe, Australia and New Zealand [3, 4]. It grows vigorously into tall, dense monodominant clumps or ‘stands’ which possess a shared underground rhizome system and spread vegetatively to expand up to several metres outwards and over a metre downwards from the parent plant [5, 6]. These stands have marked negative effects on the environment [7] including: a reduction in ecosystem services in riparian zones [8–11], the weakening of flood defences [9, 11, 12], changes in species abundance [13–16] and a diminution in property values [5, 17]. The socio-economic cost also includes the expense of control measures, usually comprising repeated treatments with the controversial herbicide, glyphosate [14].

Many hypotheses have been proposed to explain invasive success, including: fluctuating-resource-availability, enemy-release, Evolution of Increased Competitive Ability (EICA), naturalization, diversity-invasibility, novel weapons (NWH), shifting-defence, hybridisation-invasion, ecotype, plasticity [13, 18–20]. The ecotype hypothesis suggests that the ability of invasive plant species to thrive across different habitats is underpinned by genetic variations leading to local adaptations [16]. Whilst in some cases a genetic bottleneck can reduce the fitness of the resultant population, paradoxically some invasive species appear to thrive despite a reduced genetic diversity in the founder population [15]. Japanese knotweed exhibits minimal genetic variation in Central Europe [21], Norway [22] and the USA [23], and exists as a female clone in the United Kingdom from a single introduction [24, 25]. The ability of populations with low genetic diversity such as Japanese knotweed to take advantage of a wider ecological niche has been attributed to phenotypic plasticity [13, 26–32], efficient resource partitioning [33] and vegetative regeneration [34], allowing *R. japonica* to colonise a broad geographic range across diverse habitats such as riparian wetlands, urban transport courses, and coastal areas [21, 23]. However, there is also evidence that phenotypic plasticity is similar between invasive plants and native or non-invasive closely related species [35, 36]. Previous studies have suggested that invasive plants are phytochemically unique in their new habitats, conferring advantages such as antiherbivore, antifungal, antimicrobial and allelopathic effects [37]. Plant anti-herbivory and anti-pathogen defences are conferred in part by phenolic phytochemicals such as tannins, lignin and quercetin [38].

Here we have employed the technique of attenuated total reflection Fourier transform infrared (ATR-FTIR) spectroscopy combined with chemometrics to study the biomolecular adaptations of clonal *R. japonica* to growth in habitats with contrasting soil characteristics and climatic conditions. This technique provides rapid, marker-free, non-destructive analysis of biological samples [39]. Applications of ATR-FTIR studies in plants now include identification of plants from different growing regions [40–42]; plant response to abiotic factors such as soil fertility [43], heavy metals [44, 45], water and temperature stress [46], nutrient deficiency and uptake [47, 48]; as well as monitoring plant health and development [49–51] and infection [52].

ATR-FTIR works by using infrared light of wavenumbers 4000–400 cm^− 1^ (2.5–25 μm wavelengths) to induce atomic displacement and a change of dipole moment within the bonds of biomolecules [53], which preferentially absorb light of wavenumbers 1800–900 cm^− 1^, a range known as the ‘fingerprint region’. Spectral acquisition provides complex multivariate data and is therefore coupled with chemometrics. Subtle differences in sample composition can be analysed using mathematical techniques such as principal component analysis (PCA) and linear discriminant analysis (LDA), support vector machine (SVM), naïve bayes, and artificial neural networks (ANN) [54–57]. This provides biochemical information about proteins, nucleic acids (DNA/RNA), lipids and carbohydrates [58] because the absorption patterns are characteristic of the chemical composition, structure and function of the sample [59]. Associated wavenumber shifts in the ATR-FTIR spectral fingerprint have been identified for biologically significant compounds of interest such as the herbicide glyphosate [60], and the endogenous biological compounds; tannins [61], cutin [62], cutan [62], lignin [50], carotenoids [50], ellagic acid [63], and quercetin [63]. Definitions for characteristic peak frequencies commonly seen in ATR-FTIR studies have been compiled in databases and are available in the literature from previous studies, for example see [64, 65].

The process from chemometric biomarker identification to physical biomolecular extraction is a developing area of spectroscopy with an ongoing research effort currently focused on optimising the quantification of biomolecular concentrations with the resultant spectra in biological extracts [66, 67], consolidating the expanding database of key wavenumber changes and their associated molecular definitions [64], and trialling new biological applications [48, 49, 52]. Sample preparation such as freeze drying or grinding may influence the spectra acquired from vegetative plant material and the resultant classification success of subsequent chemometric analysis. To ensure optimum spectral quality and molecular sensitivity, instrumental settings and sample preparation must be adjusted prior to spectral acquisition [53, 54, 68, 69].

To gain insight into how Japanese knotweed plants respond to and colonise varied environmental habitats, we examined their spectral fingerprints using the machine learning method, SVM. Variations in the obtained spectral fingerprint region were sufficiently distinct to differentiate between plants collected from sites in North-East England, North-West England, and Scotland with high accuracy. Key wavenumber changes indicated chemical differences between growing regions in several biomolecules: the cell wall component pectin, phenolic and antioxidant compounds (including carotenoids, tannins, ellagic acid, quercetin), lipids and fatty acids, the Amide I and II regions of proteins, and the nucleobases adenine and cytosine. To correlate spectral differences with environmental data, soil was collected from each site and climatic data collected by the United Kingdom Met Office was used [70]. Regional differences in the spectral fingerprint of *R. japonica* detected by ATR-FTIR spectroscopy and SVM could not be explained by pH, water content, organic matter content, plant available phosphorus, carbon to nitrogen ratio, maximum temperature, minimum temperature, air frost, days of rain, amount of rain, or the number of days of sunlight. Future studies will identify the mechanisms that underpin the regional differences in the spectral fingerprint of *R. japonica* and which contribute plasticity to *R. japonica* allowing it to thrive in such diverse habitats.

## Methods

### Field sites

In late-summer 2018, plant and soil samples were collected from seven contrasting sites across the Northern United Kingdom where Japanese knotweed is known to be a problem (see Supplementary Table S1). Stands were identified according to their morphological features as described within the literature, see [71]. The data were then analysed by region (West Scotland [WS], North West England [NWE], North East England [NEE]) or site (Scotland [SOM, SAP, SLM, SRC], North East England [EDB], North West England [ESA, ESB]).

### Sample collection and storage

Leaves were collected from three different canes per site of Japanese knotweed. On each cane, three leaves were collected from different positions on the plant, designated the labels ‘New’, ‘Height’, or ‘Mature’. The relevant landowners were contacted for permissions to collect sample materials. The topmost newly unfurled leaf was collected, designated ‘New’. ‘Height’ leaves were collected from 1 m above the soil surface from the main cane, to account for stands of different statures. ‘Mature’ leaves were the second leaf off the first stem branching off the main cane. Interestingly, the spectral profiles were affected by leaf position, data not shown. Therefore, to ensure that developmental stage was not a confounding factor when comparing sites all three leaf positions were included in the analysis. Leaves were dried at 37 °C for 1 week and stored in a dry airtight container at room temperature before analysis using ATR-FTIR. Soil was collected from the base of each cane used in the leaf study, using a 25 cm long and 1 cm diameter bore [72]. The soil was passed through a 0.5 mm sieve and air dried before analysis [73].

### ATR-FTIR spectroscopy

Dried leaves were analysed using a Tensor 27 FTIR spectrometer with a Helios ATR attachment (Bruker Optics Ltd., Coventry, UK). The sampling area, defined by the Internal Reflection Element (IRE), which was a diamond crystal, was 250 μm × 250 μm. Each leaf was placed on a slide with the section to be analysed facing upwards; the slide was then placed on a moving platform and moved upwards to ensure a good and consistent contact with the diamond crystal. Spectral resolution was 8 cm^− 1^ with two-times zero-filling, giving a data-spacing of 4 cm^− 1^ over the range 4000 to 400 cm^− 1^; 32 co-additions and a mirror velocity of 2.2 kHz were used for optimum signal to noise ratio. In total 1260 spectra were taken, ten spectra from each side of sixty-three leaves (three leaves from each of three canes per seven sites). All spectra are available in the Supplementary Dataset. To minimise bias, an even spread of ten spectra were taken from each surface of the leaf, resulting in a total of twenty spectra per leaf. Approximately the same position on each leaf was located using a camera attachment, with five spectra taken either side of the central leaf vein.

### Spectral data handling and analysis

All spectral information was converted from OPUS format to suitable files (.txt) before input to MATLAB (Mathworks, Natick, USA). Pre-processing of the acquired spectra is an essential step of all spectroscopic experiments and is used to improve the signal-to-noise ratio by correcting problems associated with random or systematic artefacts during spectral acquisition including different sample thickness [74]. Pre-processing and computational analysis of the data were performed using an in-house developed IRootLab toolbox [39, 75] and the PLS Toolbox version 7.9.3 (Eigenvector Research, Inc., Manson, USA), according to standardised protocols for analysis of biochemical spectra [69, 76]. Spectra were cut at the biochemical fingerprint region (1800–900 cm^− 1^), Savitzky-Golay (SG) second differentiated and vector normalised. The number of points used in SG smoothing was nine. All data were mean-centred before multivariate analysis. To view the raw spectra see Supplementary Figure S1.

For the classification of groups, principal component analysis followed by linear discriminant analysis (PCA-LDA) and support vector machines (SVM) were used. PCA was used to reduce the original data into a few sets of variables called principal components (PCs). These variables, composed of ‘scores’ and ‘loadings’, are orthogonal to each other and account for most of the explained variance from the original data set. Scores were used to identify similarities and dissimilarities among the samples whilst loadings identify the weight contributed to the PCA model by each variable [42]. As PCA is an unsupervised technique, the category variables were not used for this dataset reduction. To perform a supervised classification model, the PCA scores were employed as input variables for the discriminant algorithm, linear discriminant analysis (LDA; Morais & Lima, 2018). LDA created a linear classification rule between the classes based on a Mahalanobis distance. For exploratory data-analysis this study used the composite analysis PCA-LDA as well as the non-linear classifier, SVM [77], which was additionally used for biomarker identification. The SVM classifier was used to find the classification hyperplane which provided the largest margin of separation between the data clusters. During model construction, the data were transformed into a different feature space by means of a kernel function that is responsible for the SVM classification ability. The most common kernel function, the radial basis function (RBF [78];) was used here. Correlation between spectral differences and soil traits were assessed with PCA and partial least squares (PLS) regression. The relationship between spectra and climatic conditions (maximum temperature, minimum temperature, mean temperature, hours of sunshine, days of rainfall, days of rain ≥1 mm, and days of air frost) were also evaluated by PLS regression. Cross-validation is a model validation method used to evaluate the performance of the model when applied to an unknown sample. In this study, the number of components of PCA-LDA, PLS regression, and all SVM parameters were optimized by venetian blinds (10 data splits) cross-validation. The samples’ spectra were randomly divided into a training set (70%, 882 spectra) and an external test set (30%, 378 spectra) to perform validation.

### Soil moisture content and organic matter level

For each biological replicate, two separate technical replicates were analysed. Approximately 6.5 g of air-dried soil was dried for 48 h at 105 °C in an oven and the oven-dried mass was noted to calculate the percentage soil moisture content. The soil organic matter level was subsequently calculated by loss on ignition (LOI) [79]. The oven dried soil was placed in a furnace at 550 °C for 6 hours, and the final mass noted to calculate the percentage LOI.

### C:n

In addition to calculating organic matter content by LOI, carbon and nitrogen levels were measured individually, and their values compared. Soil samples were dried overnight at 105 °C, before grinding for 2 min at 400 rpm. A microbalance was used to measure out 30 mg of dried-ground soil, which was then wrapped in tin foil boats for analysis in an Elemental Analyser (elementar vario EL III).

### Plant available phosphate

Plant available phosphate was measured using the Olsen P method [80]. This method uses bicarbonate as a chemical extractant to simulate the uptake of phosphorus by plants from the solution and exchange surfaces in soil in the form of phosphate. Three biological replicates were analysed per site. For each replicate, air-dried soil (2 g) was added to pH adjusted sodium bicarbonate (NaHCO_3_, 0.5 M, 40 mL). This mixture was placed in an orbital shaker at 200 rpm for 30 mins, before filtration with Whatman 42,110 mm filter paper. Plant available phosphorus was measured using a SEAL AA3 AutoAnalyser with a SEAL XY-2 AutoSampler. The solutions from the first six samples were measured three times to check the consistency of the machine.

### pH

Soil pH was measured based on the procedure created by Allen [81]. Soil (10 g fresh weight) was mixed with distilled mili-Q water (25 mL) for 30 min in an orbital shaker. The mixture was left in the fridge overnight to settle. The pH at the soil-water interface was then measured out using a Mettler Toledo SevenCompact™ pH meter.

### Climatic data

Met Office published climatic data were used for this study, for the regions West Scotland, North West England, and North East England, for the time period of the growing season ‘Summer 2018’ [70]. Maximum temperature, minimum temperature, mean temperature, hours of sunshine (as a measure of photoperiod), days of rainfall, days of rain ≥1 mm, and days of air frost were considered.

### Statistics

Statistical significance of measured soil parameters was calculated in R [82]. A Shapiro–Wilk test indicated non-normal distribution therefore the data were analysed using the non-parametric Kruskal-Wallis test followed by a post hoc test using the criterium Fisher’s least significant difference (LSD) within the package ‘agricolae’ [83] to determine where the difference lies between sites, signified by lowercase letters (Fig. 6a-d). Alpha was set at 0.05. Within each graph, all bars which share letters are not significantly different from each other. Graphs were produced in RStudio using the package ggplot [84].

## Results and discussion

### Pre-processing of IR spectra in the fingerprint region reveals differences between regions

**Fig. 1:**
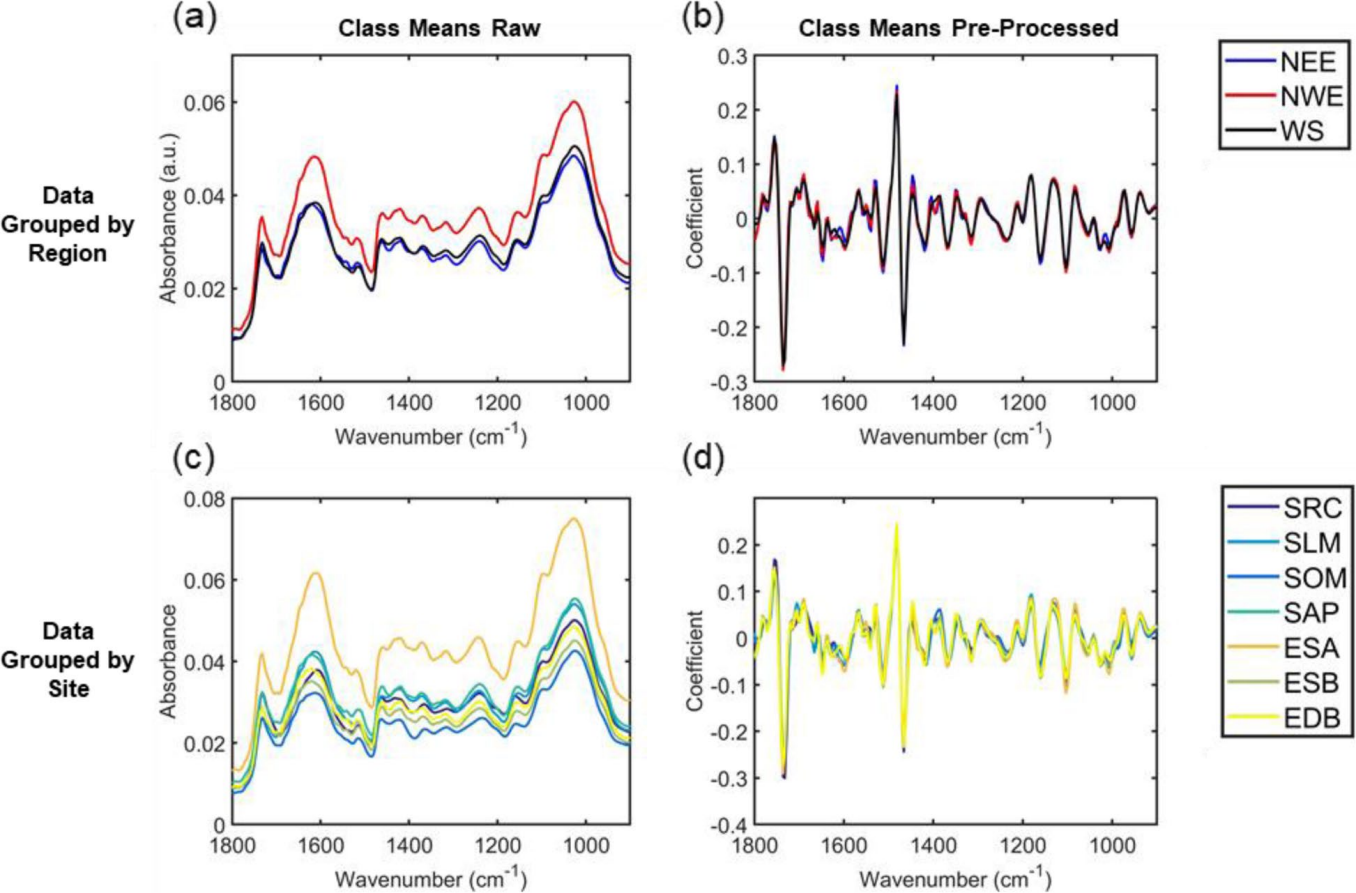
**(a)** Class means raw and **(b)** class means pre-processed (SG smoothed and vector normalised) IR spectra in the fingerprint region (1800–900 cm^− 1^) grouped by the different regions where knotweed samples were collected (NEE: North East England, NWE: North West England, WS: West Scotland); **(c)** class means raw and **(d)** class means pre-processed (SG smoothed and vector normalised) spectra in the fingerprint region (1800–900 cm^− 1^) grouped by the different sites where knotweed samples were collected (Scotland: SRC, SOM, SLM, SAP; North West England: ESA, ESB; North East England: EDB)

To capture and quantify the plant’s response to its environment, ATR-FTIR spectroscopy was used. ATR-FTIR spectra were taken from both leaf surfaces of Japanese knotweed. Figure 1 shows the raw and pre-processed spectra, where the mean spectra at the fingerprint region are depicted by region (West Scotland [WS], North West England [NWE], North East England [NEE]; Fig. 1a and b) and sites (Scotland [SOM, SAP, SLM, SRC], North East England [EDB], North West England [ESA, ESB]; Fig. 1c and d) from which they were collected. There are subtle visual differences in the mean spectra from knotweeds collected in the WS, in particular at around 1648 cm^− 1^ (Amide I band), 1586 cm^− 1^ (Amide II band) and 1400 cm^− 1^ (symmetric stretching of COO- of amino and fatty acids). Amide bands confer information on the secondary structure of proteins and are sensitive to protein conformation by differing degrees [85]. The Amide I band is the most sensitive, and originates from the C=O stretching vibration of the amide group coupled with the in-phase bending of the N-H bond and stretching of the C-N bond [86, 87]. The Amide II peak is a combination of N-H in plane bending and C-N stretching [87].. The mean knotweed spectra collected from NWE also show subtle visual differences at around 1650 cm^− 1^ and 1580 cm^− 1^.

### Knotweed from different regions are distinguishable on the basis of spectral profile

**Fig. 2:**
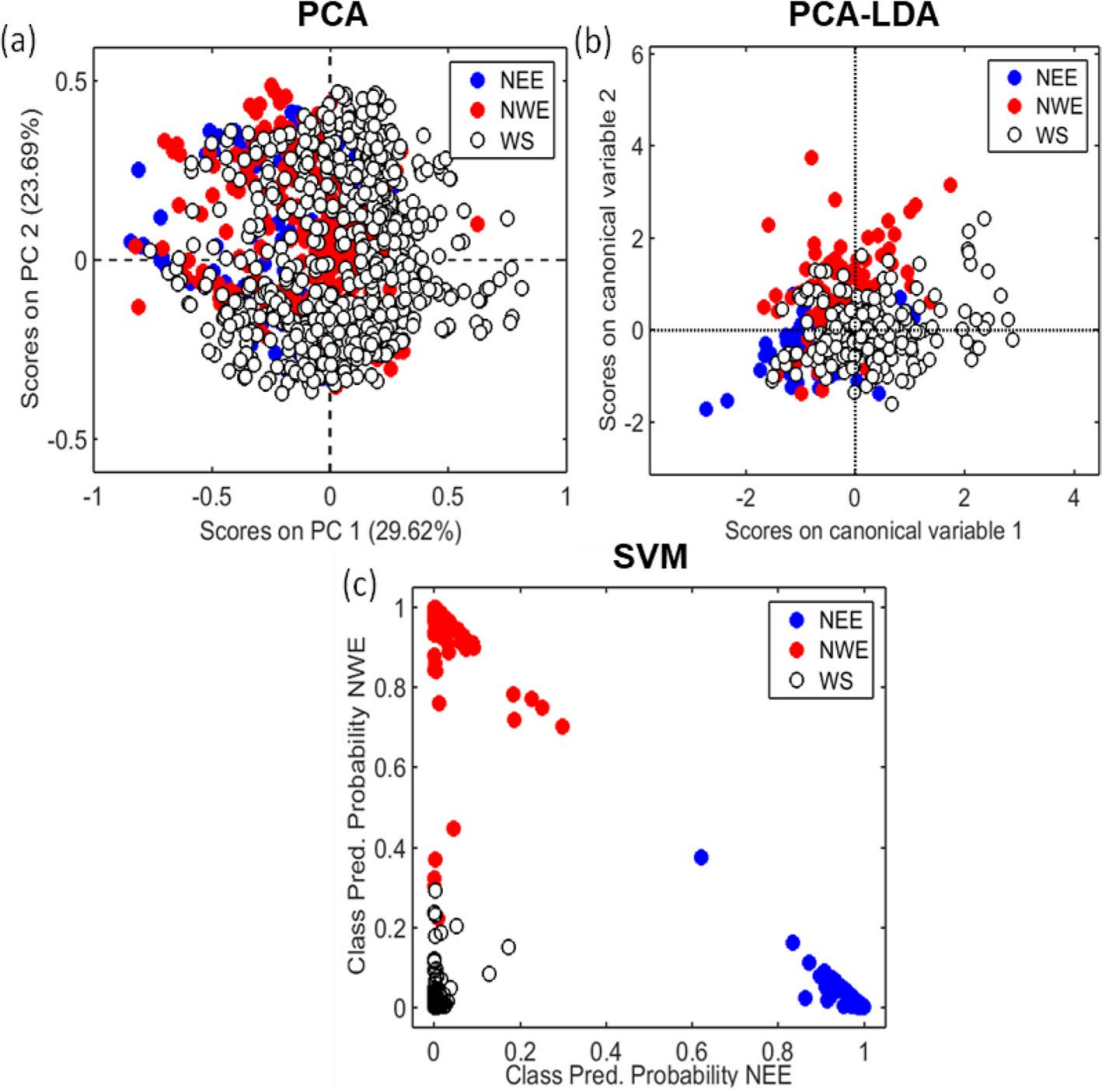
**(a)** PCA scores, **(b)** PCA-LDA canonical scores and **(c)** SVM class predicted probability for the IR spectral dataset according to different regions where knotweed samples were collected (NEE: North East England, NWE: North West England, WS: West Scotland). Numbers inside parenthesis indicate the percentage of explained variance in each PC. Each spectral point in these scores plots represents a single spectral acquisition

Changes in the spectral fingerprint region were sufficient to successfully identify sites from different geographical regions with high accuracy using SVM chemometrics, indicating that Japanese knotweed from within each region share common properties that are distinct from those of plants from other regions. This resulted in them grouping together (Fig. 2).

Unsupervised PCA was used to explore natural differences between knotweed samples collected from different regions (NEE, NWE and WS). No clear difference is observed in the scores on PC1 and PC2, indicating high similarities between the spectral profiles (Fig. 2a). This is consistent with most Japanese knotweed in the United Kingdom being a genetically uniform clone, described as a component of the “world’s largest female” in biomass terms [9]. Therefore, supervised methods of analysis, PCA-LDA and SVM, were applied to distinguish the samples based on their region. PCA-LDA was constructed using 10 PCs (93% explained variance) with a training performance of 68% accuracy (cross-validation accuracy of 67%). The predictive performance of PCA-LDA towards the external test set was relatively poor (96, 90, 50% specificity; 51, 40, 87% sensitivity; 69, 62, 70% precision for classes 1,2, and 3 respectively; Table 1), where the groups were found overlapping (Fig. 2b). SVM (cost = 10, *γ* = 3.16, *N*_*SV*_: 439) however, performed better in both training (~ 100% accuracy) and test sets (95%) (Table 1), indicating that knotweed plants can be differentiated by region based on their IR spectral profile. PCA-LDA assumes that the features of Japanese knotweed from each region are a realisation of a multivariate normal distribution, which is unlikely to be true. SVM on the other hand does not make this assumption, and given the right kernel function can uniformly approximate the true boundary between the classes of knotweed from each region by the universal approximation theorem [88].

**Table 1:** Quality parameters for spectral classification based on different regions. The predictive performance of PCA-LDA towards the external test set was relatively poor. However, SVM performed well in training and test sets, indicating that knotweed leaf samples can be differentiated by region based on their IR spectral profile

Algorithm	Class	Accuracy	Sensitivity	Specificity
PCA-LDA	North East England	63%	30%	96%
	North West England	62%	37%	87%
	West Scotland	67%	88%	47%
SVM	North East England	100%	100%	100%
	North West England	98%	95%	100%
	West Scotland	98%	100%	97%

**Fig. 3:**
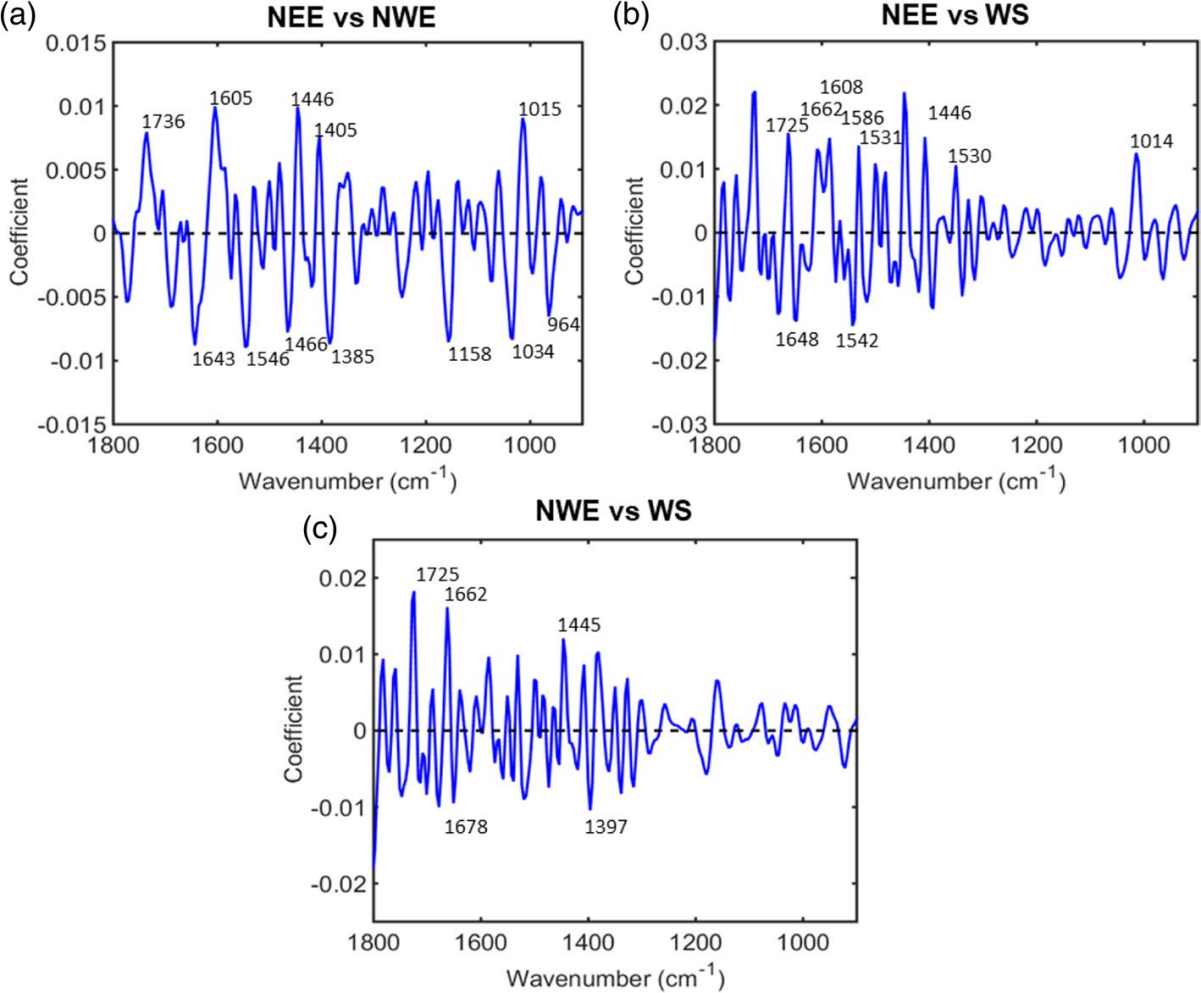
Difference between mean support vector spectra for **(a)** North East England (+ coefficients) and North West England (− coefficients), **(b)** North East England (+ coefficients) and West Scotland (− coefficients), **(c)** North West England (+ coefficients) and West Scotland (− coefficients). The main wavenumbers responsible for class differentiation between the three regions are labelled, and have been used to identify spectral markers

Examination of the difference-between-mean support vectors spectra found by SVM revealed spectral markers indicative of marked chemical differences between Japanese knotweed from different regions. Figure 3 shows the main wavenumbers responsible for class differentiation between the three regions which, through comparison with the literature, have been used to identify spectral biomarkers (Table 2). This indicates that there are marked chemical differences between Japanese knotweed from Scotland, North West England and North East England. Differences between regions were identified at wavenumbers 1736, 1643, 1605, 1546, 1466, 1446, 1405, 1385, 1158, 1034, 1015, 964 cm^− 1^ between NEE and NWE; 1725, 1662, 1648, 1608, 1586, 1542, 1531, 1446, 1530, 1014 cm^− 1^ between NEE and WS; 1725, 1678, 1662, 1445, 1397 between NWE and WS cm^− 1^ (Table 2). These are indicative of differences in the cell wall component pectin, phenolic and antioxidant compounds, lipids and fatty acids, the Amide I and II regions of proteins, and the nucleobases adenine and cytosine between knotweed from different regions.

**Table 2:** Biomolecular markers for class differentiation between the three regions (Scotland, North West England and North East England) and each site (SOM, SAP, SRC, SLM, ESA, ESB and EDB). Spectral markers were found by investigating the difference-between-mean support vectors spectra found by SVM, and linked to the biomolecules associated with each wavenumber from published literature

Comparison	Wavenumber/ cm^**−1**^	Tentative Molecular Assignment	Reference
NEE and NW	1736	C=O stretching [lipids]	[65]
	1643	C=O stretching [Amide I]	[65]
	1605	*v*_as_ (COO^−^) [polysaccharides, pectin]	[65]
	1546	Amide II: [protein N–H bending, C–N stretching], α-helical structure	[89]
	1466	CH_2_ bending in lipid	[50]
	1446	aromatic ring stretch vibrations, tannins	[61]
	1405	CH_3_ asymmetric deformation	[65]
	1385	Ring stretching vibrations mixed strongly with CH in-plane bending	[65]
	1158	*v*C-O of proteins and carbohydrates	[65]
	1034	C-O stretch, tannins	[61]
	1015	ν (CO), ν (CC), δ (OCH), ring in pectin	[50]
	964	C-O deoxyribose, C-C	[65]
NEE and WS	1725	C=O stretching band mode of the fatty acid ester	[65]
	1662	Amide I, or fatty acid esters	[65]
	1648	Amide I	[65]
	1608	aromatic ring stretch vibrations, tannins	[61]
	1586	Amide II	[65]
	1542	Amide II	[90]
	1531	Amide II	[91]
	1446	aromatic ring stretch vibrations, tannins	[61]
	1530	C=N adenine, cytosine	[65]
	1014	phosphodiester stretching bands [symmetrical and asymmetrical]	[65]
NWE and WS	1725	C=O stretching band mode of the fatty acid ester	[65]
	1678	Stretching C=O vibrations that are H-bonded [changes in the C=O stretching vibrations could be connected with destruction of old H-bonds and creation of the new ones]	[65]
	1662	Amide I, or fatty acid esters	[65]
	1445	lipids	[63]
	1397	CH_3_ symmetric deformation	[65]
SRC and others	1748	C=O stretching vibration of alkyl ester, pectin	[62]
	1728	ν (C=O) ester, cutin	[62]
	1678	Stretching C=O vibrations that are H-bonded [changes in the C55O stretching vibrations could be connected with destruction of old H-bonds and creation of the new ones]	[65]
	1651	phenolic compounds/ cutan [aromatic and C=C functional groups]	[62]
	1608	aromatic ring stretch vibrations, tannins	[61]
	1542	Amide II	[90]
	1455	C-O-H	[65]
	1443	*δ* (CH_2_) [lipids, fatty acids], or *δ*(CH) [polysaccharides, pectin]	[65]
SLM and others	1755	lipid	[51]
	1735	C=O stretching, the phenolic compound ellagic acid/ the secondary metabolite quercetin	[63]
	1512	ν (C-C) aromatic (conjugated with C=C phenolic compounds	[62]
	1481	symmetric deformation NH_2_^+^, glyphosate^X^	[60]
	1466	CH_2_ bending in lipid	[50]
SOM and others	1755	lipid	[51]
	1736	lipids	[63]
	1481	symmetric deformation NH_2_^+^, glyphosate^X^	[60]
	1466	CH_2_ bending in lipid	[50]
	1161	carbohydrate; stretching vibrations of hydrogen-bonding C–OH groups (found in serine, threonine and tyrosine residues of cellular proteins); cellulose	[51]
	1103	*ν*(C–O–C) in ester	[50]
SAP and others	1755	lipid	[51]
	1736	lipid	[63]
	1481	symmetric deformation NH_2_^+^, glyphosate^X^	[60]
	1466	CH_2_ bending in lipid	[50]
	1103	*ν*(C–O–C) in ester	[50]
ESA and others	1755	lipid	[51]
	1732	lipid; fatty acid esters; hemicellulose	[51]
	1647	amide I; pectin	[51]
	1512	ν(C=C) in lignin, carotenoid or protein	[50]
	1481	symmetric deformation NH_2_^+^, glyphosate^X^	[60]
	1466	aromatic ring stretch vibrations, tannins	[61]
ESB and others	1755	lipid	[51]
	1736	C=O stretching [lipids]	[65]
	1481	symmetric deformation NH_2_^+^, glyphosate^X^	[60]
	1466	CH_2_ bending in lipid, or aromatic ring stretch vibrations, tannins	[50, 61]
EDB and others	1728	ν (C=O) ester, cutin	[62]
	1446	aromatic ring stretch vibrations, tannins	[61]
	1408	CH_3_ deformation, *ν*_s_ (COO^−^) in pectin	[50]

Two of the key peaks for differentiation between NEE and NWE regions, 1015 and 1605 cm^− 1^, were linked to pectin. The rubber-band corrected spectra indicated that the pectin concentration was lowest for NEE at both peaks compared with other regions. A horizontal shift in the SG differentiated spectra for NEE at both peaks indicate an altered pectin structure for North East samples. This could be of interest as manipulation of pectin synthesis is often studied, due to the compound’s importance in food products and biofuel production [92]. Two peaks corresponding to tannins, 1034 cm^− 1^ and 1608 cm^− 1^, were responsible for the differences between regions, NEE vs NWE and NEE vs WS respectively.

The concentration of tannins is higher in WS and NWE than in NEE, indicated by the higher absorbance levels in the rubber-band corrected spectra. A biochemical structural change in tannins at NEE is exhibited, indicated by a horizontal shift at both 1034 cm^− 1^ and 1608 cm^− 1^ in the SG differentiated spectra. This would suggest that the reduced tannin levels of Japanese knotweed at the site in North East England would make it a more favourable host for herbivorous biocontrol agents [38].

### ATR-FTIR spectral changes allowed discrimination on a site-by-site basis

**Fig. 4:**
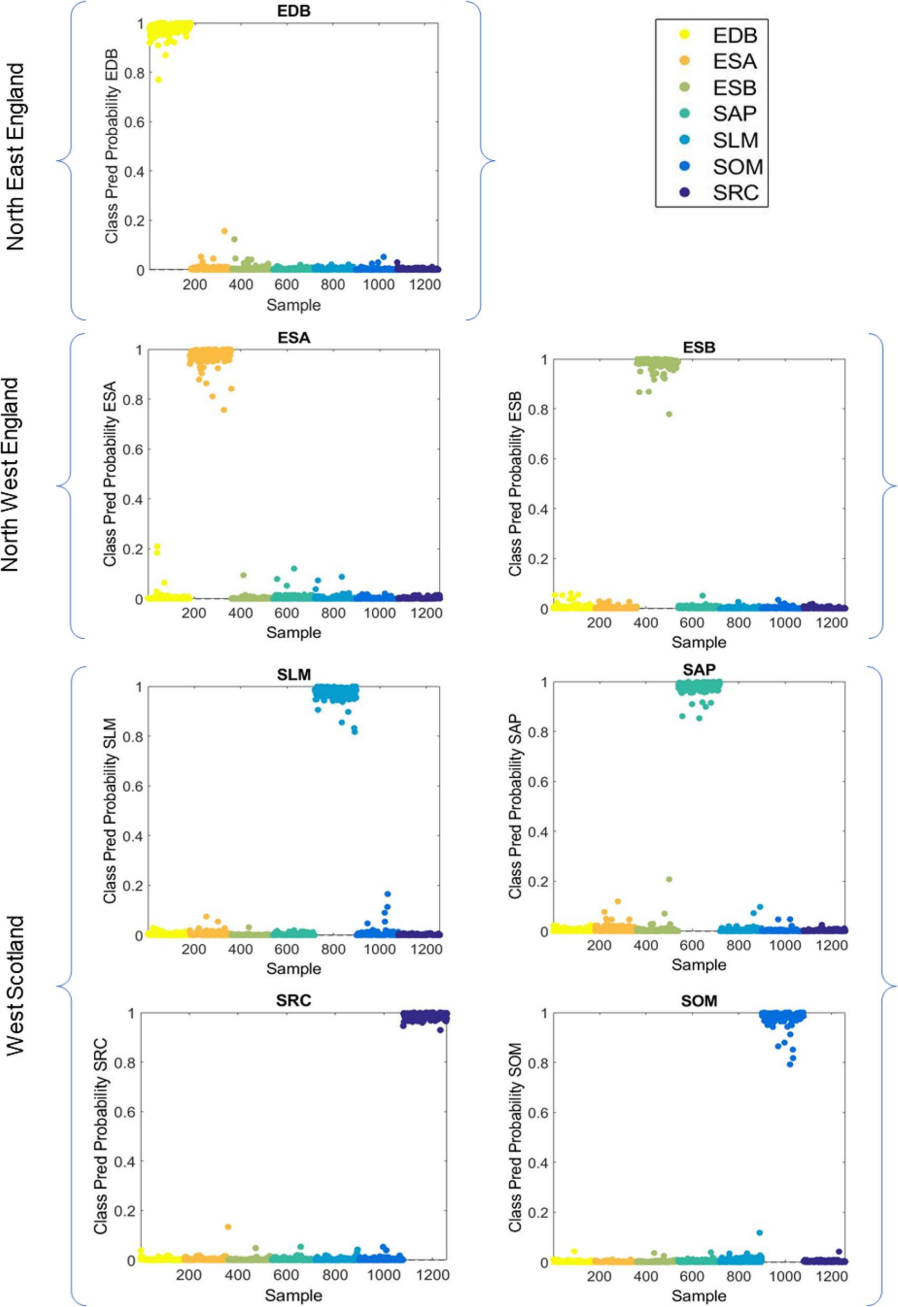
SVM class predicted probability for the IR spectral dataset according to different sites where knotweed samples were collected (Scotland: SRC, SOM, SLM, SAP; North West England: ESA, ESB; North East England: EDB). The clear separation indicates that the knotweed samples can be differentiated by the site at which they were collected

In addition to investigating interregional variations between knotweed from different geographical areas, each site colonised by Japanese knotweed was further investigated individually to see how varying environmental conditions in each habitat affected the plant’s spectral fingerprint. Due to the highly satisfactory classification performance of SVM, site differences were also investigated using this method. To view the PCA scores plots see Supplementary Fig. S5. SVM was trained using cost = 10, *γ* = 3.16; *N*_*SV*_: 675, generating accuracies at 100% for training and 94% for class validation. The predictive capability of SVM was tested in an external test set where accuracies were found ranging from 97 to 100%, sensitivities from 94 to 100%, and specificities from 99 to 100% (Table 3), indicating that the knotweed samples can be differentiated by the site at which they were collected. Figure 4 shows the SVM class predicted probability for the IR spectral dataset on a site-by-site basis.

**Table 3:** Quality parameters for spectral classification based on different sites. Separation by PCA-LDA was relatively poor. However, SVM performed much better, indicating that knotweed leaf samples can be differentiated by the site at which they were collected using this method

Algorithm	Class	Accuracy	Sensitivity	Specificity
PCA-LDA (10 PCs, 91% explained variance)	SRC	85%	80%	90%
	SLM	70%	57%	83%
	SOM	60%	24%	97%
	SAP	62%	35%	89%
	ESA	73%	52%	95%
	ESB	56%	20%	92%
	EDB	65%	43%	88%
SVM	SRC	100%	100%	100%
	SLM	100%	100%	100%
	SOM	99%	98%	100%
	SAP	100%	100%	100%
	ESA	100%	100%	100%
	ESB	97%	94%	100%
	EDB	99%	100%	99%

**Fig. 5:**
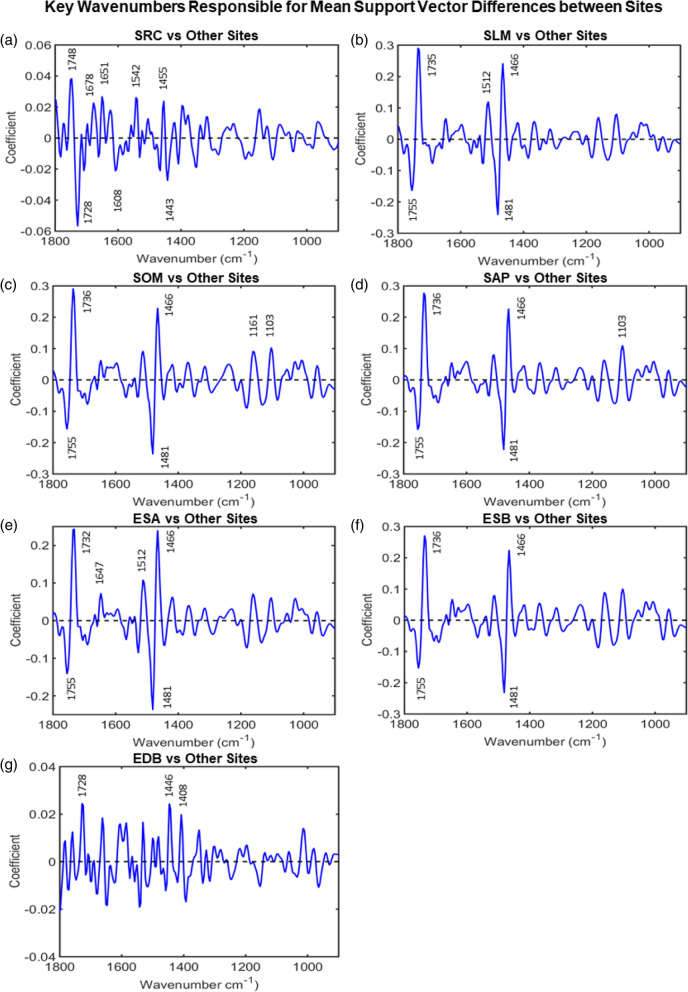
Difference between mean support vector spectra for **(a)** SRC (+ coefficients) and others (− coefficients), **(b)** SLM (+ coefficients) and others (− coefficients), **(c)** SOM (+ coefficients) and others (− coefficients), **(d)** SAP (+ coefficients) and others (− coefficients), **(e)** ESA (+ coefficients) and others (− coefficients), **(f)** ESB (+ coefficients) and others (− coefficients), and **(g)** EDB (+ coefficients) and others (− coefficients). These comparisons can be used to identify the key wavenumbers responsible for the differences between sites, which have been labelled above, and can be used to find spectral biomarkers

Using the same method described above, the difference between mean support vector spectra of a site was compared with the other six sites, for each site in turn. These comparisons were used to identify the key wavenumbers responsible for the differences between sites (Fig. 5). The spectral markers associated with site difference were at wavenumbers: 1748, 1728, 1678, 1651, 1542, 1455, 1443 cm^− 1^ for SRC and others; 1755, 1735, 1512, 1481, 1466 cm^− 1^ for SLM and others; 1755, 1736, 1481, 1466, 1161, 1103 cm^− 1^ for SOM and others; 1755, 1736, 1481, 1466, 1103 cm^− 1^ for SAP and others; 1755, 1732, 1647, 1512, 1481, 1466 cm^− 1^ for ESA and others; 1755, 1736, 1481, 1466 cm^− 1^ for ESB and others; 1728, 1446, 1408 cm^− 1^ for EDB and others, (Table 2). The changes in spectral profile were associated with the prominent cuticle components, cutan and cutin, and the cell wall component, pectin. While cuticle components were key for site-by-site classification, these were not used for regional distinction. Site-by-site classification required spectral biomarkers strongly associated with phenolic antioxidant compounds, including carotenoids, tannins, ellagic acid, quercetin. Other key identifiers included lipids, fatty acids, and the Amide I and II vibrational modes of proteins.

Five of the seven sites showed traces of treatment with glyphosate: EDB, ESA, SAP, SOM and SLM. Three of the sites, ESB, SOM, and SAP, appeared dead the following summer, indicating that they had received an effective dose of glyphosate the previous year. Upon collection at site SLM, the plants were noted to appear in good health, and despite showing traces of herbicide in ATR-FTIR spectroscopy studies, plants at the site were still alive and well the following year. The herbicide history of NEE and WS sites is unknown, however, ESA and ESB are known to have been treated the year prior to sample collection. Repetitive herbicide use is commonplace in Japanese knotweed removal, with a minimum of 3 years glyphosate treatment being the most common chemical treatment method. Jones et al. [14]*,* found that repeated glyphosate use was the most effective control method. However, sub-lethal herbicide doses are known to have a hormetic effect on plants [93], with the potential to increase vigour of target plants and arm them against other stresses. Therefore, prior sub-lethal herbicide treatment may have resulted in altered gene expression being reflected in the spectral biomarkers in certain sites.

A family of membrane-stabilising plant pigments called carotenoids were flagged up as a biomarker for site ESA. Carotenoids participate in light-harvesting and are essential for photoprotection against excess light [94]. It is therefore surprising that they would appear as a key identifier of a shaded woodland site like ESA. However, biomarkers for both glyphosate (at 1481 cm^− 1^), and carotenoid (at 1512 cm^− 1^), presented as differences between ESA and the other sites. Carotenoid levels are known to be affected by herbicide application, including a temporary increase in response to reactive oxygen species, followed by a decrease due to reduced biosynthesis [95–97].

The presence of the biomarker for quercetin (at 1735 cm^− 1^) is consistent with current knowledge of this plant species [98, 99]. Quercetin is a flavonoid with antioxidant properties which also acts as a naturally occurring auxin polar transport inhibitor [100]. The marker at 1608 cm^− 1^ has been linked to tannins in the quebracho tree, and the marker at 1446 cm^− 1^ is present in several tannins [61].

### Soil analysis indicates the environmental diversity between sites

As a clonal species which spreads by physical disturbance of rhizome, crown and stem propagules, Japanese knotweed occupies habitats where disturbance occurs, such as near roads, railways and water courses [101]. Although all the sites from which Japanese knotweed was collected share these characteristics, the environmental conditions and habitats colonised by this species were variable within each region. For example, within Scotland there were two riverside sites with adjacent forest: a brownfield site repurposed as a park which had historically been farmed, built on, mined, quarried, dug for clay and used as a landfill site and a railway siding; in addition to an urban site adjacent to a railway line, road, and public footpath (see Supplementary Table S1 for site descriptions, Google Maps coordinates, and photographs).

**Fig. 6:**
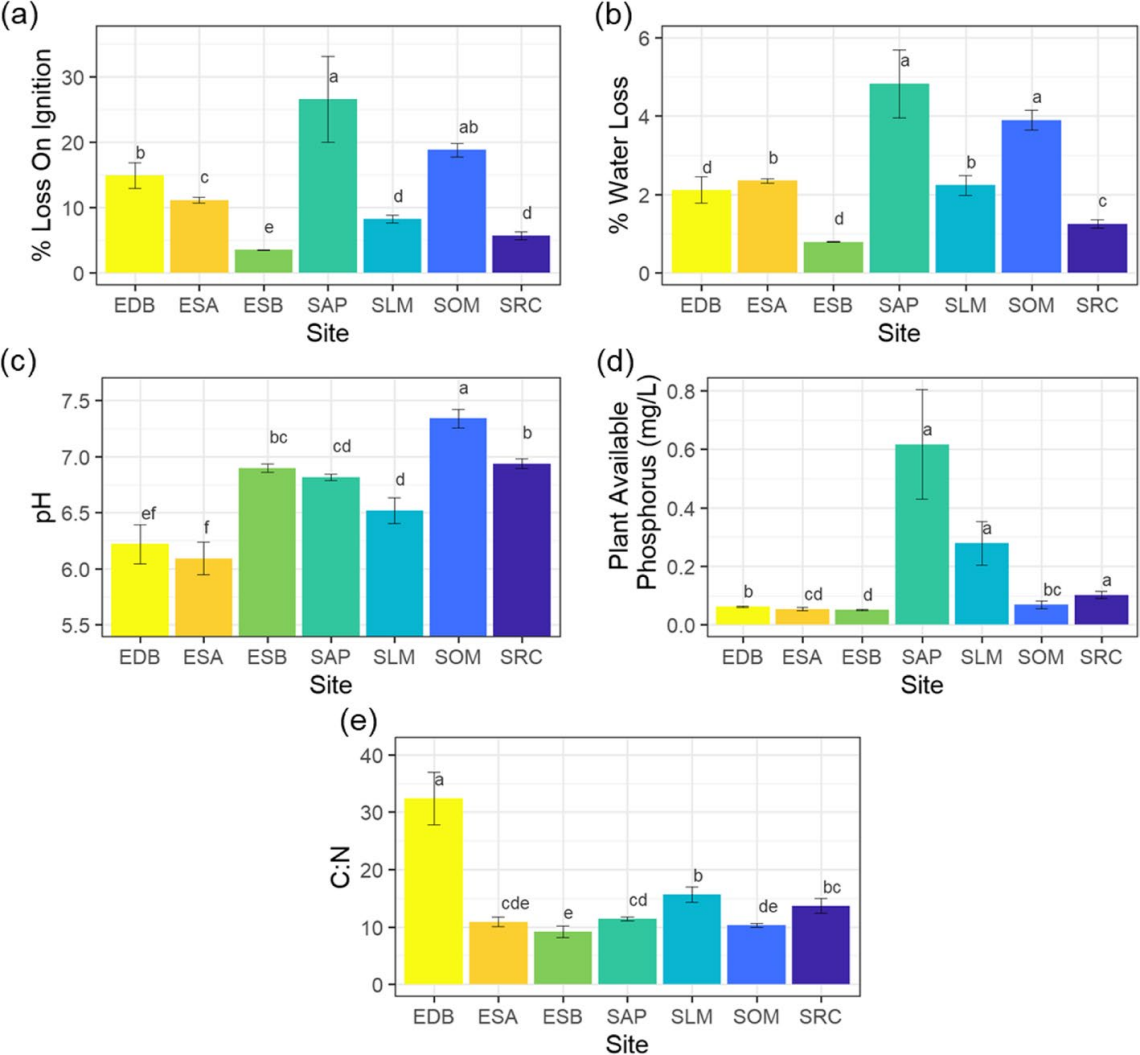
Soil parameters for each site, with error bars showing standard error; a) percentage mass lost on ignition (LOI), b) % water loss, c) pH, d) plant available phosphorus, e) carbon to nitrogen ratio (C:N). Statistical significance was calculated using a Kruskal-Wallis followed by a post hoc test using the criterium Fisher’s least significant difference (LSD) to determine where the difference lies, signified by lowercase letters above the bars. Within each graph, all bars which share letters are not significantly different from each other. Data are mean +/− standard errors. pH, *n* = 9; C:N n = 9; % water loss mean, *n* = 6; % loss on ignition mean, n = 6; plant available phosphorus mean, *n* = 3 except for EDB and ESB where n = 9

**Fig. 7:**
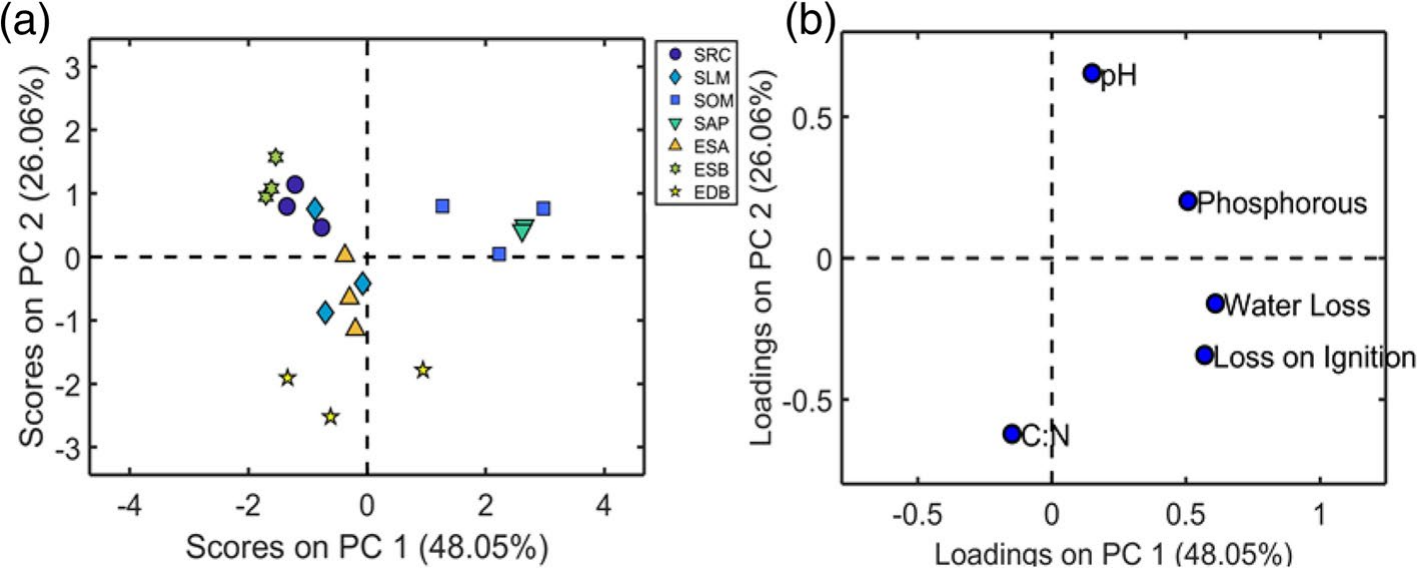
**(a)** PCA scores and **(b)** loadings for soil data (abbreviations define sites where samples were collected, Scotland: SRC, SOM, SLM, SAP; North West England: ESA, ESB; North East England: EDB). The North East England soil sample, EDB, has a high C:N ratio, and a lower pH than the other samples. EDB soil was found to be naturally different from the others on the PCA scores plot, with a greater separation in the Y-plane (PC2). ESB has a higher C:N than the other sites. SOM and SAP have high phosphorus, water loss, and LOI (organic carbon) compared with SRC, SLM, ESA, ESB, and EDB. SLM was a mixed sample, sharing similar soil traits with ESA and SRC. Note: An extreme sample was removed. SAP3 was a non-homologous urban environment and one of the three soil samples was an outlier. The bar graphs in Fig. 6a and b show high standard deviation for loss on ignition and percentage water loss for SAP, due to this sample

This environmental diversity between sites is reflected in differences in measured soil characteristics; considered individually in Fig. 6, and in combination through the multivariate analysis shown in Fig. 7. The two related graphs shown in Fig. 7 are the PCA scores (7a) and PCA loadings (7b) plots for the relationship between the chemical soil parameters measured at each site. Overall, Fig. 7a shows a segregation pattern which indicates site-by-site differences in measured soil traits. The three points of each category in the scores plot represent the three biological replicates collected from each site, their proximity to one another is an indication of variation within the site. The distance of scores away from the origin in Fig. 7a show how different the sites were from one another, and the explanations for their separation can be found by looking at the loadings in Fig. 7b. Scores which are close to a loading point have a higher value of this parameter. The trajectory of the samples can be thought of as a modulus, because a negative score or loading can still mean a higher value in terms of the soil trait. For example, the North East samples, EDB, which are closer to the C:N loading area had a higher C:N ratio than the others despite being in the negative region of the graph.

EDB soil was found to be naturally different from that of the other sites on the PCA scores plot, with a greater separation in the Y-plane (PC2). Although both Figs. 6 and 7 agree that EDB had a higher C:N ratio compared with the other sites (nearly double), when considered individually in Fig. 6e it is clear from the error bars that this high level varied between samples, whereas the lower ratios for SAP and SOM were more consistent between samples. Nitrogen is vital for the synthesis of chlorophyll, nucleic acids and proteins [102] and the C:N ratio is a good measure of decomposition rates, with higher C:N ratios generally leading to longer decomposition rates. Figure 7 highlights that ESB had a lower C:N than the other sites, suggesting that this riverside site in full sun had the fastest decomposition rate of all the sites.

In addition to a high C:N ratio, Fig. 7 shows that EBD also had a lower pH than the other samples. This is consistent with Soilscapes’ definition of this area as having ‘slowly permeable seasonally wet acid loamy and clayey soils’, with low fertility and impeded drainage (UK Soil Observatory, 2020). Soil pH affects nutrient availability and in clay soils pH may alter the structure of the soil; pH in the range 5.5 to 6.5 is normally optimum for allowing plants access to most nutrients [103]. The pH was higher in SOM compared with all other sites (Fig. 6c). The pH of SRC and ESB were significantly higher than SLM, EDB and ESA, with SRC additionally higher than SAP (Fig. 6c). SAP has a mid-range pH which is similar to SLM but higher than EDB and ESA. EDB and ESA have the lowest pHs, although there was variability between samples (Fig. 6c). In general, phosphorus availability decreases with increasing pH, however the multivariate analysis placed these loadings within the same quadrant, connecting instead a high C:N ratio with a low pH. Low pH soil reduces the growth of the bacteria and fungi responsible for the breakdown of organic matter and nutrient cycling. As a species which produces abundant recalcitrant polyphenol-rich leaf litter [7, 104], Japanese knotweed may therefore be expected to have a more detrimental effect on the nutrient cycling in acidic soil sites such as EDB and ESA. However, only EDB exhibited this carbon accumulation.

Overall, SOM had the highest pH, organic matter, and water content, with little variability between samples. In Fig. 7, PC1 separates SOM and SAP whilst PC2 separates the other samples. The loadings show that SOM and SAP have high phosphorus, water loss, and LOI (organic carbon) compared with SRC, SLM, ESA, ESB, and EDB. Although collected in a similar region to the mineral gleys of SLM and SRC, SOM and SAP were both urban sites with anthropic soils. Soil organic matter can improve the soil’s water holding capacity, enhances aggregate stability, increases cation exchange capacity, acts as a nutrient source and alters the soil microbiome [105]. The site with the lowest organic matter content, ESB, is significantly different from the other sites (Fig. 6a). Sites SLM and SRC have lower organic matter levels than all sites except ESB (Fig. 6a). ESA had a mid-range organic matter content which is different from the other sites (Fig. 6a). SOM has a similar organic matter content to both EDB and SAP. The site with the highest organic matter content, SAP, had a high level of variability between the samples (Fig. 6a).

The water content of EDB and ESB was lower than the other sites. SRC soil contained the next highest level of water, followed by ESA and SLM, followed by SAP and SOM which have the highest (Fig. 6b). Overall ESB, a clay-to-sandy loam soil [106] collected from a site on the River Wyre had both low organic matter and water contents with minimal variation between samples. The PCA in Fig. 7 shows that SLM was a mixed sample, sharing similar soil traits with ESA and SRC. This grouping may be surprising as site ESB had a more comparable riverside location to SLM and SRC. Despite their differing proximity to water, the soils of ESA, SRC, and SLM were all a similar cohesive clay-like consistency whereas the soil of riverside site ESB was much sandier in texture. Additionally, the surrounding vegetation was similarly well-populated by trees at sites ESA, SRC and SLM, presumably providing similar levels of cover and nutrient deposition from leaf litter.

Phosphorus (P) is essential for both ATP and nucleic acids formation [102] and often limits plant productivity because of its low mobility in soil. ESA and ESB had similar levels of plant available phosphorus, whilst ESB was lower than the other sites, ESA was similar to SOM, which in turn is similar to EDB (Fig. 6d). SAP, SLM and SRC had significantly higher plant available phosphorus values compared with the other sites (Fig. 6d), although the variation between samples in SAP was much greater. One possible reason for the enhanced phosphorus content of soil from site SAP is its proximity to an urban footpath likely to contain dog excrement, which would support the high variability within the site of plant available phosphorus. When all five soil parameters are compared at once using PCA multivariate analysis to detect outlying data [107], one of the SAP samples appears as anomalous in terms of high values for organic matter and water content (Supplementary Figure S3). This is consistent with SAP being a non-homologous urban environment located between a footpath and a railway embankment with a nearby road, thereby being prone to contamination with organic matter, and partial shade. The spectral data for SAP3 concur with the anomalous nature of this sample, as does the variability shown in Fig. 6a, b, and d. Consequently, this outlier was removed from subsequent multivariant analyses (Fig. 7a). This outlier effect supports that the bar graphs for loss on ignition and percentage water loss for SAP display a high standard deviation due to this sample.

### Soil and climatic conditions do not explain the spectral differences between site

Chemometric analysis of ATR-FTIR spectral data shows that Japanese knotweed can be identified on a site-by site basis, but that, despite this intra-regional variation, enough commonality is present within a whole region to allow discrimination of samples on a regional-basis. This raises the question of what environmental stimulus causes the variation in functional groups detected by ATR-FTIR spectroscopy? In order to investigate this, spectral data and soil data were considered together (see Supplementary Fig. S2 for PCA scores of spectral and soil data combined). The main climatic difference between the three regions was higher rainfall in West Scotland, see Supplementary Fig. S4. Partial least squares (PLS) regression of the soil characteristic data climatic data (maximum temperature, minimum temperature, mean temperature, hours of sunshine, days of rainfall, days of rain ≥1 mm, and days of air frost) from the Met Office records for Summer 2018 [70] revealed only a minimal correlation between spectral differences and soil traits (Supplementary Figs. S6a-e) or climatic conditions (Supplementary Figs. S7a-e). Therefore, the chemical differences between Japanese knotweed from NWE, NEE and WS could not be explained by the soil or climatic parameters measured in this study.

Allelopathy, releasing chemicals into the soil to alter its characteristics (Murrell et al., 2011) for example to alter nutrient availability in the rhizosphere, could provide an explanation for the absence of any correlation. Root-mediated localised acidification of the rhizosphere and soil microbes can have a marked effect on plant available phosphorus. Allelopathic plants, such as Japanese knotweed and other weed species, are particularly good at altering their soil environment [108–110]. It is standard practice to collect soil samples at one of two depths: 7.5 cm for grassland, or 25 cm depth for agricultural fields [72]. A 25 cm depth was chosen for this study, although the extensive rhizome system of Japanese knotweed spans much deeper than this (often up to 2 m below the surface [111]. Therefore, the measured depth of topsoil may not have been representative of the soil environment experienced by a well-established site of Japanese knotweed, with an interconnected rhizome system. Furthermore, Japanese knotweed demonstrates stronger allelopathic effects in artificial soils which have greater aeration, water retention, permeability, and nitrogen content and lower bulk density [108], adding a greater level of complexity to the interaction of these plants with their habitats. Allelopathy in Japanese knotweed is thought to be subject to resource allocation and is increased when nutrient supply is high [108], inhibiting the growth rather than the germination of native species [109]. This plant species is a known opportunist, which is able to take advantage of the fluctuation in resources associated with riparian areas [112] using its superior nitrogen-use efficiency compared with native species [113]. Plasticity is also thought to be resource dependent, with invasive species showing greater phenotypic plasticity than native plants if resources are plentiful [35].

Additionally, rapidly growing plant species often promote nutrient cycling displaying exploitative traits such as high tissue nitrogen content and specific leaf area, because these plants input high-quality resources to the soil [114, 115]. Japanese knotweed can increase nutrient cycling, with the greatest impact on sites which if uninvaded would have low nutrient levels. Sites occupied by Japanese knotweed can increase topsoil concentrations of exchangeable nutrients when compared with nearby uninvaded sites; Cu: + 45%, K: + 34%, Mg: + 49%, Mn: + 61%, P: + 44%, Zn: + 75% [116] possibly due to the deep extensive rhizome system allowing extraction of nutrients which are not easily accessible to other vascular plants [117]. In addition to altering the nutritional value of its soil environment, Japanese knotweed can also capitalise on available resources when they arise. In fact it performs best when nutrients come in waves rather than at a consistent level [112]. This adaptability and the fluctuating nutrition of water-side sites may explain the minimal correlation between the soil measurements and the ATR-FTIR spectral data derived from vegetative tissues.

### Differences between regions may result from phenotypic plasticity

ATR-FTIR spectroscopy with subsequent chemometric analysis has proven effective at differentiating Japanese knotweed from different geographical regions, despite this plant being considered clonal in the United Kingdom. Phenotypic plasticity, where one genotype can express different phenotypes, could be an explanation for the ability to identify plants from different regions, despite their supposable genetic consistency. This is particularly significant for clonal plants, such as Japanese knotweed [118]. Phenotypic plasticity is a potentially important mechanism for introduced species in overcoming the genetic bottleneck, maintaining health components such as growth, survival, fertility and overall vigour [16, 29–31]. Populations of alien plants are known to have higher frequencies of clonality than native plant species [119], and clonality is thought to be an important characteristic of invasive alien plants [118, 120]. A high proportion of successful invasive plants are clonal; 70% of 468 studied species from the ICUN database and 81% of the one hundred worst invasive plants [118]. Epigenetics could be an important mechanism for clonal plants as by reproducing asexually they are able to bypass the meiotic resetting of epigenetic modifications [118]. Asexual species can additionally maintain genetic variation through somatic mutation, allowing adaptation to changing environmental conditions [118, 121]. Epigenetic modifications in gene expression and function have been recognized as key mechanisms behind phenotypic variation of plant traits in response to environmental cues [18, 122]. Although there are plastic responses which are not epigenetic such as provisioning and biochemical functioning [118, 123–127], phenotypic variation of plant traits in response to environmental cues could be a result of epigenetic modifications to gene expression and function [18, 122]. This raises the intriguing possibility that epigenetic modifications may contribute to the phenotypic plasticity allowing successful invasion of Japanese knotweed in a diverse range of habitats [21, 23, 32]. In Western Europe, very little genetic variation of Japanese knotweed has been found [21, 25], which is consistent with the lack of dramatic variation shown in the PCA results in this study (Fig. 2a). However, the ability to separate Japanese knotweed spectra with SVM (Fig. 2c) indicates that there are differences common to each region. Despite its clonal nature, AFLP studies have shown an unprecedented level of epigenetic variation in *R. japonica*, particularly across Central Europe [21, 23]. Additionally, in North America invasion of diverse habitats by few Japanese knotweed genotypes has been correlated with epigenetic differentiation, with the conclusion that some epigenetic loci may respond to local microhabitat conditions [23]. Japanese knotweed from different sources grown in the same greenhouse have been known to possess differing levels of physiological vigour, with the French variant growing more vigorously than its Japanese counterpart, suggesting either a rapid evolution or pre-adaptation [128].

## Conclusion and future work

Japanese knotweed can colonise a wide variety of environmental habitats despite its genetic continuity as the world’s largest female clone. ATR-FTIR spectroscopy with subsequent chemometric analysis proved to be a successful tool for identifying Japanese knotweed grown in different environments, and even individual sites within the same geographical region. However, the chemical differences between Japanese knotweed from NWE, NEE and WS could not be explained by the soil or climatic parameters measured in this study. This lack of correlation raises important questions about the causes of these subtle variances because, as revealed by ATR-FTIR spectroscopy, subtle differences do exist between regions. These variations may be due to phenotypic plasticity, a trait shared by other clonal invasive plants. Further studies will be necessary to elucidate the mechanistic basis for the effects of environmental conditions on Japanese knotweed, including the possible contribution of epigenetic modifications, and the connection with the robust growth habit of this species.

## Supplementary Information

**Additional file 1.**

**Additional file 2.**

## Data Availability

The datasets generated and analysed during the current study are available in a supplementary folder.

## Abbreviations

ANN: Artificial Neural Network
ATR: Attenuated Total Reflectance
FTIR: Fourier Transform Infrared
IAS: Invasive Alien Species
IR: Infrared
IRE: Internal Reflection Element
LDA: Linear Discriminant Analysis
PC: Principal Component
PCA: Principal Component Analysis
SVM: Support Vector Machine
