# Supplementary material for: Regional differences in clonal Japanese knotweed revealed by chemometrics-linked attenuated total reflection Fourier-transform infrared spectroscopy
Source: BMC Plant Biol. 2021 Nov 9;21:522. doi: 10.1186/s12870-021-03293-y (PMC8579538; doi:10.1186/s12870-021-03293-y)
Supplement: Supplementary file 1 — Additional file 1. [file 12870_2021_3293_MOESM1_ESM.pdf]

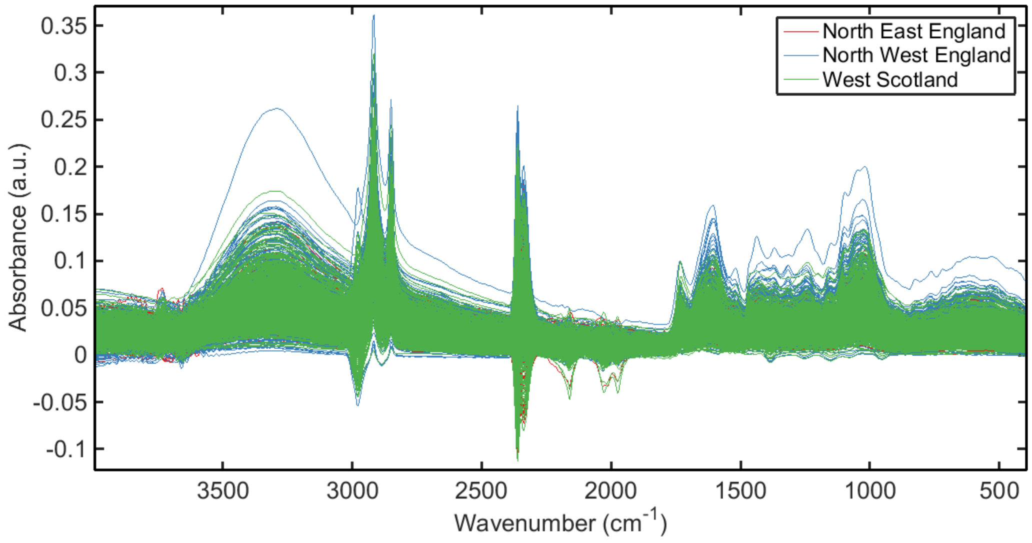

Figure S1: Raw IR data for different regions (North East England, North West England, West Scotland).

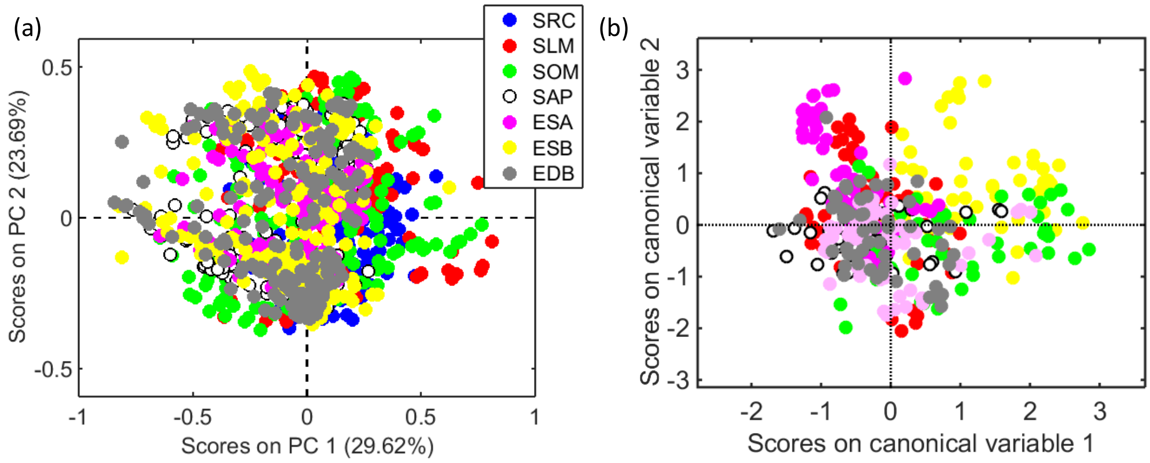

Figure S2: (a) PCA scores plot and (b) PCA-LDA canonical scores for the pre-processed spectral data in the fingerprint region for different sites where knotweed were collected (Scotland: SRC, SOM, SLM, SAP; North West England: ESA, ESB; North East England: EDB).

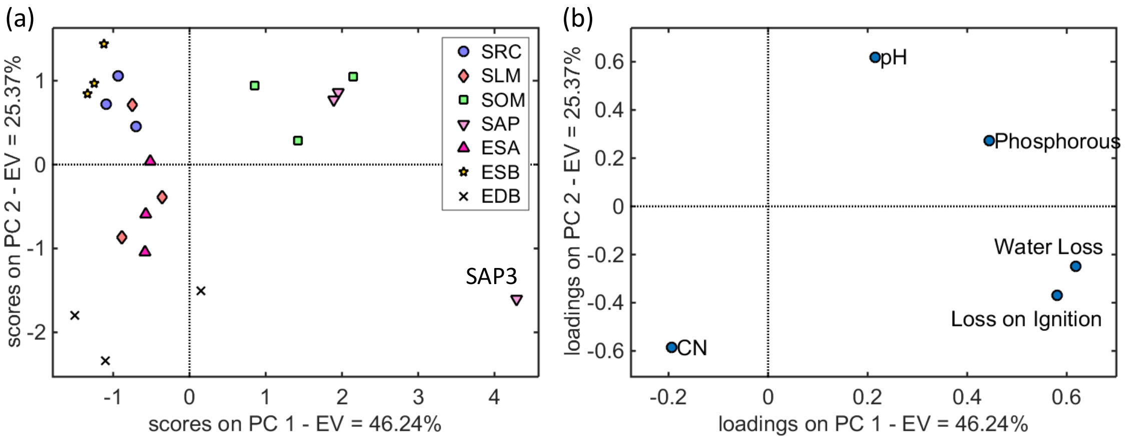

Figure S3: (a) PCA scores and (b) loadings for the soil data with extreme sample (SAP3).

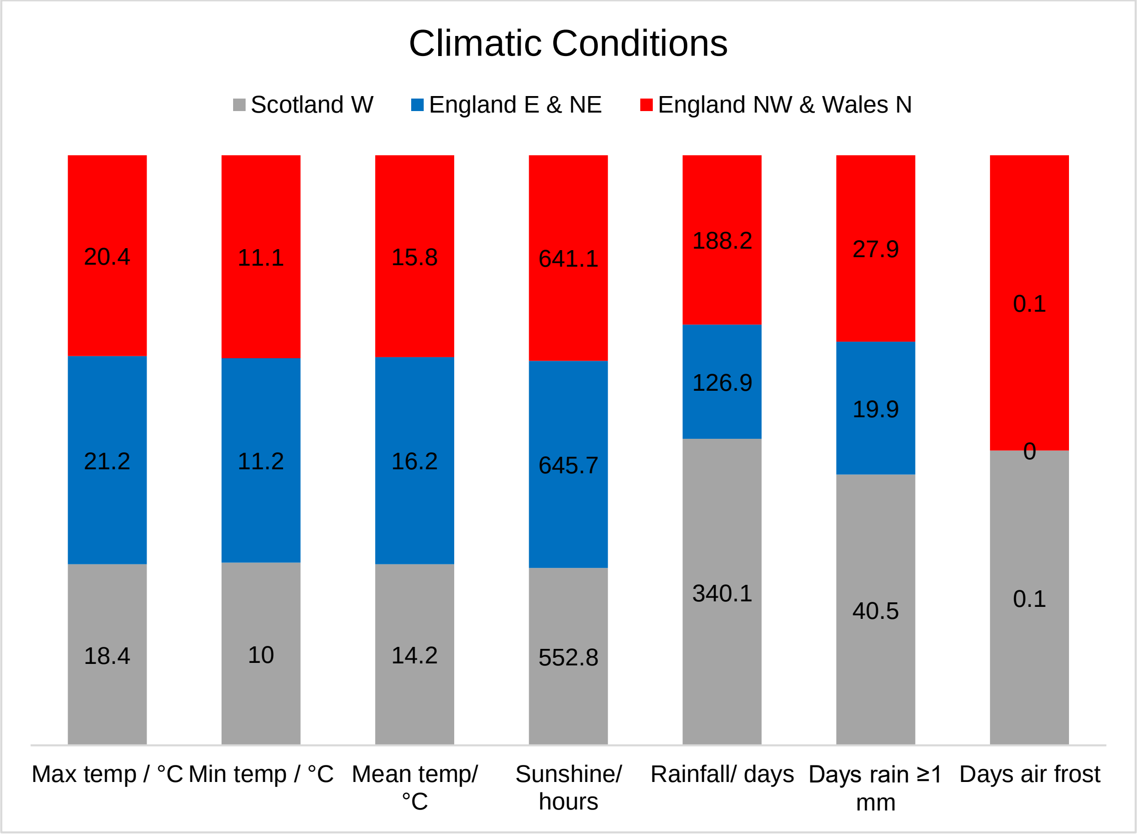

Figure S4: Climatic conditions for each region

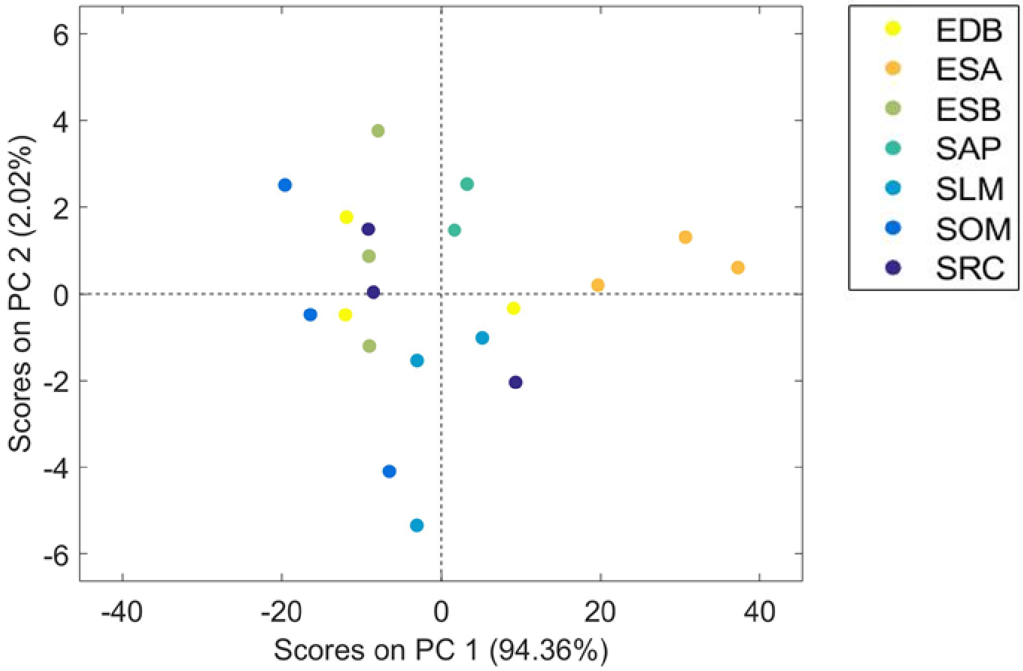

Figure S5: PCA scores for spectral and soil data combined (block-scaling applied before PCA).

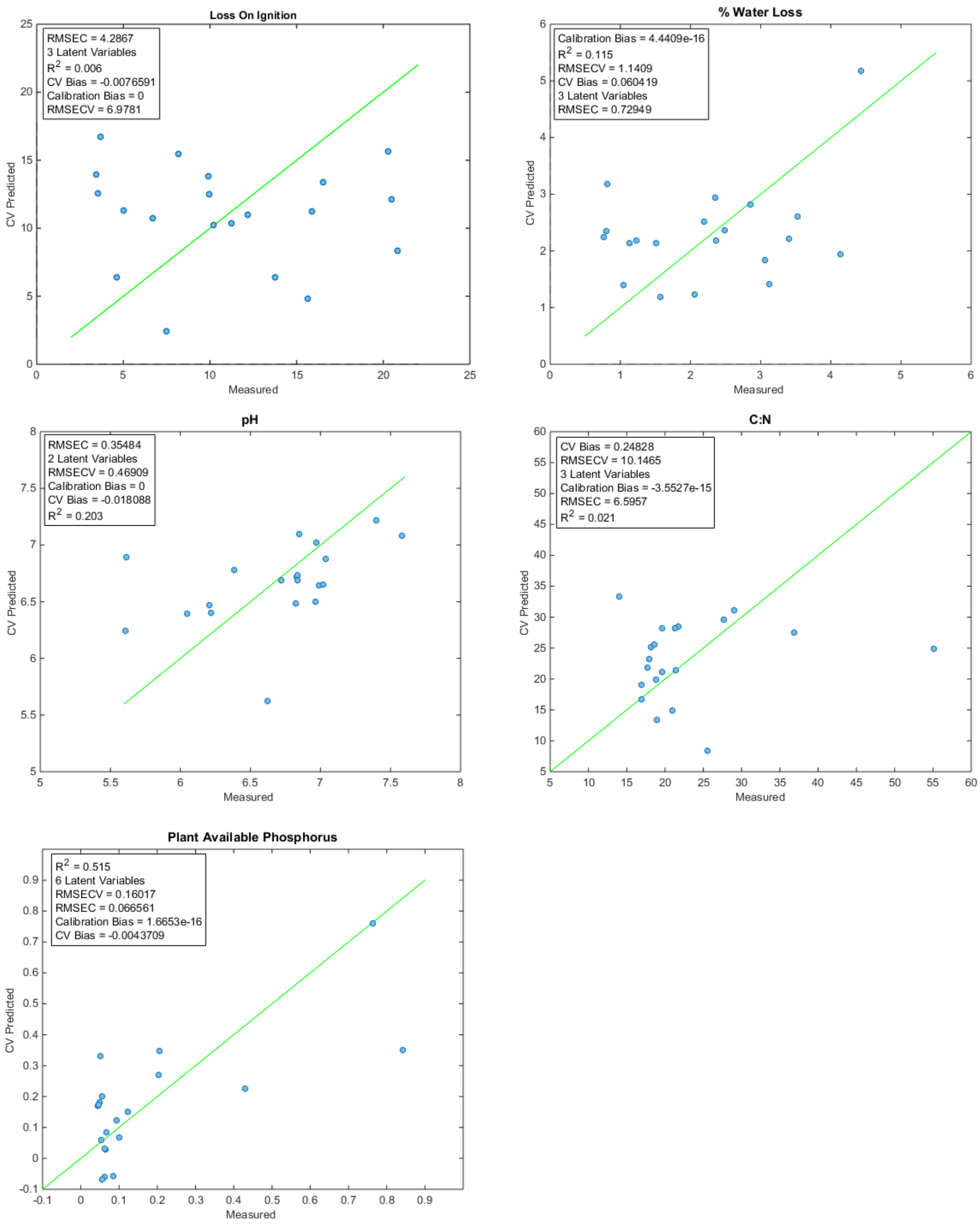

Figure S6: Measured versus predicted soil parameters based on the pre-processed spectral data using leave-one-out cross-validated partial least squares (PLS).

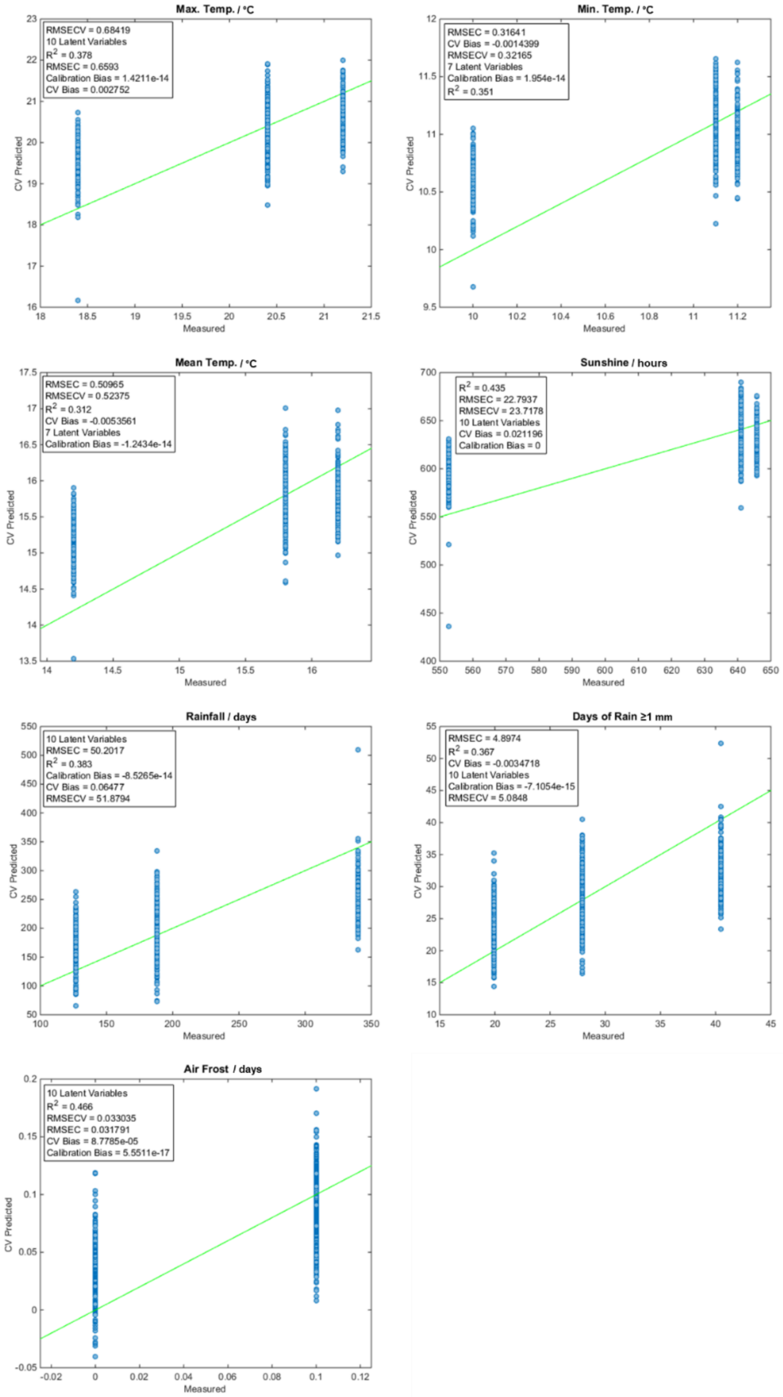

Figure S7: Measured versus predicted meteorological parameters based on the pre-processed spectral data using leave-one-out cross-validated partial least squares (PLS).

Table S1: Site Descriptions. Images taken by Claire Holden.

Stand	Description	Soil Type	Google Map Co-ordinates	Photo
**SAP**	Between a public footpath and a railway line in an urban area, near a road. Dead the following year.	Urban	55.8236264, -4.0916304	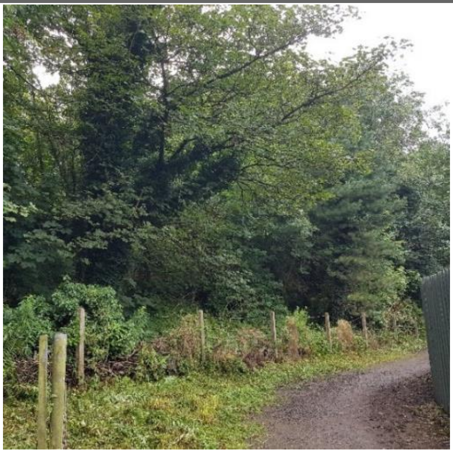
**SRC**	In full sun on Eastern bank of River Clyde	Mineral gleys	55.8186110, -4.0944440	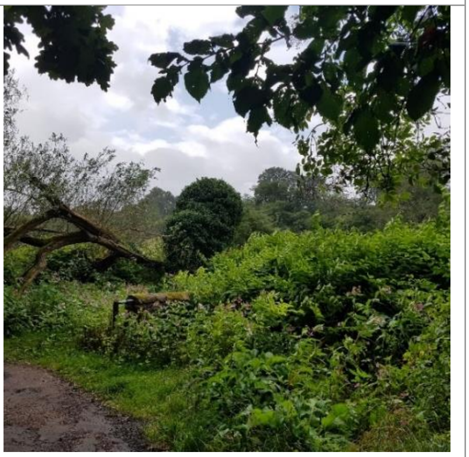
**SLM**	Western bank of River Clyde. Noticeably smaller leaves than those of SRC but appeared in overall better health	Mineral gleys	55.8026350, -4.0905780	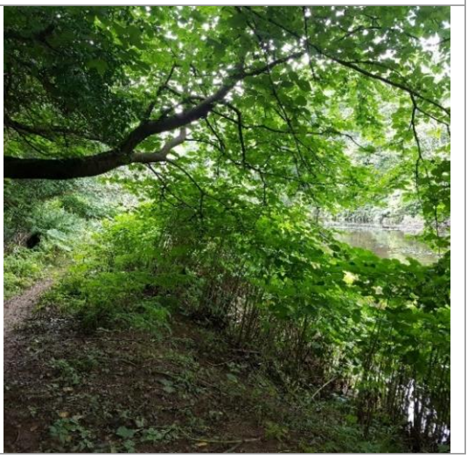

**SOM**	Brownfield site, previously used for numerous purposes including a railway siding and an old mine, but now transformed into a public park. Dead the following year.	Urban	55.8211260, -4.0554206	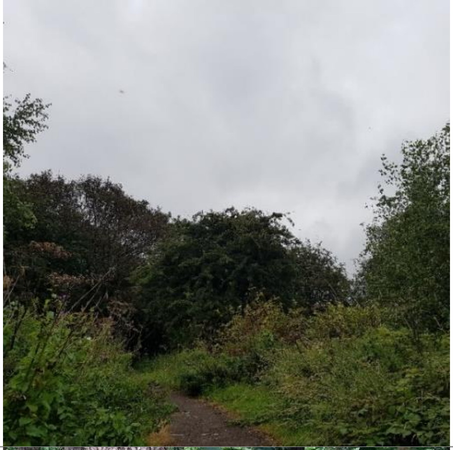
**ESA**	Wooded area further removed from river and path than ESB	Clay-to-sandy loam	53.9491670, -2.7550000	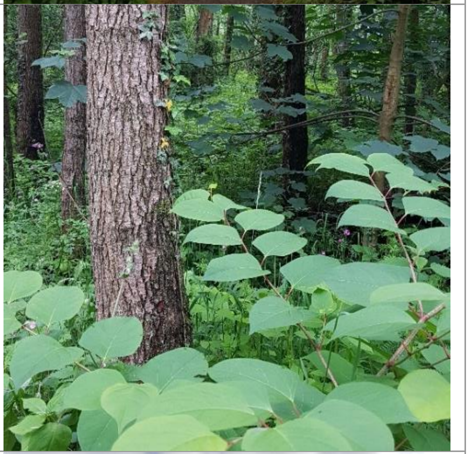
**ESB**	Edge of River Wyre. Main stand in full sun, however accessible leaves collected from the shaded side. Dead the following year.	Clay-to-sandy loam	53.94977780, -2.75541670	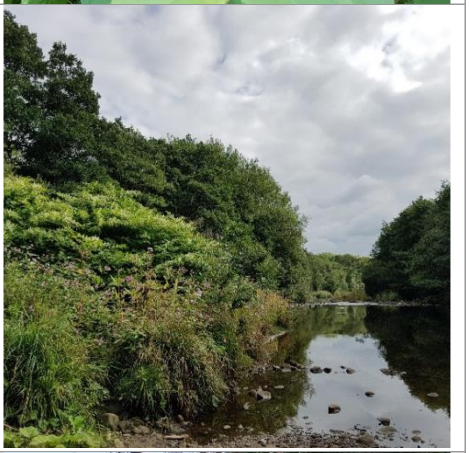
**EDB**	Adjacent to a wooded stream on the edge of one of the repurposed old railway lines now part of the Broompark cycle path network.	Slowly permeable seasonally wet acid loamy and clayey soils	54.764993, -1.609166	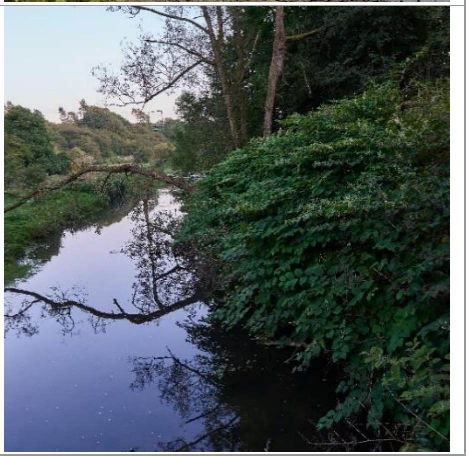
